# Maintaining a sense of normality with the help of others: Lived experiences of facilitators and barriers to Lupus adjustment

**DOI:** 10.1177/13591053241296190

**Published:** 2024-11-22

**Authors:** Sofia Silva-Ribeiro, Sónia F Bernardes, Marta M Marques, Cristina A Godinho

**Affiliations:** 1 Centre for Psychological Research and Social Intervention (CIS-Iscte), Iscte-University Institute of Lisbon, Lisbon, Portugal; 2 NOVA National School of Public Health, Public Health Research Centre, Comprehensive Health Research Center, CHRC, NOVA University Lisbon, Lisbon, Portugal

**Keywords:** chronic illness, illness adjustment, quality of life, qualitative methods, systemic lupus erythematosus

## Abstract

Systemic lupus erythematosus (SLE) has a high detrimental impact on individuals’ quality of life. Identifying key factors associated with SLE adjustment is crucial for intervention development, yet there is no previous research exploring the perspectives of individuals with SLE regarding illness adjustment’ facilitating or hindering factors. In this qualitative study, 16 individual semi-structured interviews with Portuguese adults with SLE (13 women) were conducted to explore perceived facilitators and barriers to illness adjustment. A reflexive thematic analysis revealed that efforts toward maintaining a normal life, optimism, keeping engaged in meaningful activities, accessing reliable illness-related information, and having supportive relationships were core facilitators of SLE adjustment. Main barriers included inability to maintain normality, engaging in excessive activity, pessimism, being unsupported or stigmatized, and not having access to reliable illness-related information. These findings unveil potential targets for psychosocial and behavioral interventions aimed at enhancing the quality of life for individuals with SLE.

## Introduction

Systemic lupus erythematosus (SLE) is an autoimmune disorder, which can affect almost any tissue/organ with various musculoskeletal, cutaneous, and/or hematologic manifestations (Kapoor and Mahadeshwar, 2020). Women represent 90% of SLE cases, especially those of childbearing age, and the worldwide prevalence rates range from 20 to 70 cases per 100,000 individuals (Arnaud and Van Vollenhoven, 2018). SLE bears a heavy toll on individuals’ physical functioning, psychological wellbeing (Sutanto et al., 2013). It often hampers individuals’ ability to maintain their daily (work) activities (Morgan et al., 2018), can lead to postponing parenthood due to fear of complications, and threaten individuals’ identities by being socially stigmatized (Sutanto et al., 2013).

Despite the potentially disabling impact of SLE, individuals’ illness adjustment levels vary greatly. According to the Framework of Adjustment to Chronic Diseases (Moss-Morris, 2013), achieving good illness adjustment implies reporting good quality of life and the ability to effectively manage the illness/treatments, keep reduced levels of psychological distress, maintain positive affect and minimize illness interference on life roles/relationships. Some individuals with SLE achieve good illness adjustment by staying optimistic, socially integrated, and learning effective illness self-management strategies, whereas others report lower quality of life, feeling worthless, hopeless, holding negative illness representations and seeing their life roles/projects interrupted (Petrocchi et al., 2022; Sutanto et al., 2013). So, it is important to develop evidence-based psychosocial interventions to promote better illness adjustment and quality of life of individuals with SLE (Zhang et al., 2012).

An essential step for the development of evidence-based psychosocial interventions to promote SLE adjustment is to identify its main underlying (modifiable) risk and protective factors (Moss-Morris, 2013; Sutanto et al., 2013). A recent systematic review and meta-analysis investigating the psychological, behavioral, and social factors associated to SLE adjustment showed that illness/treatment-related beliefs, social support, stress and sedentary behavior are important targets for future interventions (Silva-Ribeiro et al., 2022). Nevertheless, this systematic review only included quantitative studies, which were mainly driven by theory and researchers’ perspectives. The development of interventions to promote individuals’ illness adjustment should also be grounded on their needs and lived experiences (Yardley et al., 2015).

The lived experiences of individuals with SLE have been previously considered in qualitative research to examine their perspectives about health care provision (Hale et al., 2006), to support intervention development and evaluation (e.g. Twumasi et al., 2020), to understand the impact of a specific type of lupus (e.g. cutaneous lupus; Yan et al., 2021), or the impact of lupus on specific life domains (e.g. work; Ubhi et al., 2021) and to explore illness representations (Goodman et al., 2005). Although ample attention has, therefore, been given to several dimensions of the lived experiences of individuals with lupus, to our knowledge, no previous studies have yet explored the perspectives of individuals with SLE regarding the factors that facilitate or hinder their illness adjustment. The aim of this qualitative study was to explore the perspectives of individuals with SLE on the main facilitators and barriers to illness adjustment, conceptualized according to the Framework of Adjustment to Chronic Diseases by Moss-Morris (2013), as to subsequently inform the development of an evidence- and person-based digital intervention to promote good SLE adjustment.

## Method

### Study design and participant recruitment

This study was based on a qualitative methodology (Bradshaw et al., 2017). A convenience sampling method was used. Ten participants were recruited via healthcare professionals, other seven through social media. To be eligible for the study individuals had to: (a) be over 18 years; (b) have a SLE diagnosis; and (c) be fluent in Portuguese. One participant was excluded because she reported living with other chronic conditions with more intense and disabling manifestations than SLE, rendering SLE signs and symptoms difficult to disentangle from the signs and symptoms of the other health conditions. This study was approved by the ethics committee of Iscte-University Institute of Lisbon.

### Data collection

Sixteen individual semi-structured interviews were conducted between May 24 and June 4, 2021, in Portugal, by a trained psychologist (SR). Due to the restrictions imposed by the COVID-19 pandemic, most (*n* = 14) interviews were conducted (and audio recorded) via Zoom or WhatsApp. Two interviews were conducted by telephone, as participants were not familiar with digital platforms. After providing oral informed consent to participate in the study, participants were first asked to complete an online survey (via Qualtrics) assessing sociodemographic (i.e. age, sex, nationality, education, and income) and clinical information (i.e. age at diagnosis, presence of other chronic conditions).

The interviews were semi-structured, including three sections (Supplementary File 1): (1) ice-breaker questions, to explore the subjective experience of receiving an SLE diagnosis and of disease progression; (2) main questions, to explore the impact of SLE on individuals’ lives and, their subjective perspective on the main facilitators and barriers to SLE adjustment; (3) questions regarding the needs and preferences regarding a digital intervention to improve SLE adjustment. The third section, which is out of the scope of the present paper, was included because the present study emerges in the context of a wider research project aiming to develop a person-based digital intervention to promote SLE adjustment. We used probing questions, when needed, to clarify and further explore participants’ experiences. At the end of the interview, all participants were thanked and debriefed. Interviews lasted, on average, 40 minutes, most of them were between 30 minutes and 1 hour and 40 minutes. Four interviews lasted less than 30 minutes (*M* ≅ 19 minutes), because the interviewees reported low disease activity and illness interference on quality of life. These interviews were included in the analysis to ensure sampling diversity.

The audio recordings were transcribed verbatim by SR and four research assistants. All information that could potentially identify participants, such as names or specific locations, were removed or coded (e.g. using alias) to maintain anonymity.

### Data analysis

Braun and Clarke’s (2019, 2022) six-step methodology for reflexive thematic analysis was followed. The research team adopted an experiential perspective, assuming a critical realist and contextualist stance (Braun and Clarke, 2022). The first step of the analysis corresponded to a deep immersion in the data corpus, that is, all data produced by the interviews (Braun and Clarke, 2006). Afterward, the data set for this study was identified, that is, the parts of the data that provided an answer to our research question (Braun and Clarke, 2006). The second step corresponded to the development of the initial codes, mainly an inductive process of marking out, naming and defining significant parts of the data. A deductive approach to coding was also used, by taking into consideration previous theoretical concepts. For example, the bipolar continuum of the illness adjustment construct influenced the development of themes that reflect bipolar dimensions. Also, the research team previously carried out a systematic review to find factors associated with adjustment to lupus, influencing to some extent the way the data was interpreted and coded. A semantic coding process was privileged over latent coding. Third, an interpretative, reflexive and collaborative process took place to develop themes. The interpretative process was enriched by the diverse and complementary perspectives of the three authors directly involved in the data analysis (SR, CG, and SB). The fourth step entailed a critical review and refinement of the generated themes ensuring that they were representative of the data set. Until this step, all the material was analyzed in Portuguese, with English being used thereafter to name and define themes, to facilitate reporting. In the fifth step, themes were named and provided detailed definitions that expressed the research team interpretations of the experiences and viewpoints of the participants. Finally, in the sixth step, a written narrative was developed.

### Quality of data analysis

The data analysis procedures were guided by the four criteria outlined by Lincoln and Guba (1995) to assess the trustworthiness of qualitative research. *Credibility* was ensured using a reflexive team approach. SR conducted the interviews and created a first version of the coding framework, then the process involved the active contribution of CG and SB in developing the themes (Noble and Smith, 2015). The three authors (SR, CG, and SB) have different backgrounds and knowledge about SLE allowing the team to have different perspectives of the data. While SR was diagnosed with SLE 13 years ago, CG is a health psychologist interested in developing theory-based interventions for health promotion through behavior change and SB is a social and health psychologist interested in the role of psychosocial influences on chronic illness adaptation. *Dependability* was insured by a rigorous documentation and verification of the codes and themes assigned to the data, performed first independently and then through a discussion among the three authors (SR, CG, and SB). The principle of *confirmability* was ensured by keeping detailed records of anonymized interview transcripts, decision-making and records, keeping illustrative quotes supporting each code and theme. Finally, *transferability* was addressed by detailing the process of data collection and analysis, and offering a comprehensive description of participants and findings.

## Results

### Participants

Out of the 16 included participants, 3 were cis men and 13 were cis women. Participants were aged between 24 and 55 years (*M* = 39.63 years, *SD* = 8.33). The age at diagnosis ranged from 15 to 48 (*M* = 27.56 years, *SD* = 10.15), two participants had SLE onset during adolescence. Most participants (*n* = 13) were graduated and worked full time (*n* = 9). Two were students, and one was unable to work because of SLE. Seven participants reported having other chronic conditions (e.g. rheumatoid arthritis, hypertension). A table providing an overview of participants’ sociodemographic and clinical characteristics can be found in Supplementary File 2.

### Reflexive thematic analysis

Four overarching themes were developed reflecting main facilitators and barriers to SLE adjustment. All themes illustrate bipolar dimensions representing a continuum between factors that promote good versus bad SLE adjustment. Two themes (Theme 1 and 2) were clearly more present in participants’ speeches, thus being deeper and more complex than the other two themes.

#### Theme 1. Difficulties versus focus on maintaining a “normal life” through symptom management

The *focus on maintaining activities* and routines such as work, physical and leisure activities, social roles (e.g. parenting) despite pain or fatigue played a protective role. Perceiving control over a flare and its impact on daily life was key to providing a *sense of normality*, which was referred several times by participants, either as something they wished or fought for.

Stress was one of the factors referred as disruptive because of its’ potential to cause a flare. Almost all participants (except one), expressed that feeling distressed, worried, or anxious (with work, school and/or family affairs) could increase SLE activity and the likelihood of experiencing flares, being a barrier to adjustment. For most participants, the effect of stress was described as short-term, but for a few it could take some days, or was seen as a major trigger for the onset of SLE and its activity. A few individuals tried to explain the stress-SLE activity link via poorer sleep quality and/or an increased tendency to eat unhealthy food. Surprisingly, although *stress was seen as the biggest enemy* of SLE, no specific coping strategies were shared by participants.

Many individuals stated the usefulness of being alert and actively “*listening to the body”* to identify SLE manifestations, and, consequently, adjust their behaviors to manage them and maintain a normal life. Few individuals mentioned they could act before symptoms increased (e.g. take SOS medication). They also referred that it was vital to recognize when they could no longer manage it on their own and needed (professional) help. When “red flags” were identified (e.g. increases in pain, fatigue, rashes), a diverse range of adaptive *behavioral coping strategies* were mentioned, such as, changing medication, dieting, exercising, resting, avoiding sun exposure, asking for help and contacting the doctor, as exemplified by Ema (aged 38 years):

*“Over the years, I started to learn a little bit about how the disease works, what makes it worse or better, and so I made some small changes both in terms of food and physical exercise, avoiding sun exposure and resting more. I started to learn these tricks that help, that make all the difference, and now it*’ *s easier.”*

Of all the mentioned symptom management strategies, avoiding sun exposure was the most often mentioned, as sun exposure was clearly seen as a risk factor for SLE flares. So, participants tried to reduce or regulate sun exposure, with more restricted measures when the disease was active, which ranged from avoiding sun exposure altogether to using sun protective materials, looking for shadows, and using sunscreen.

Although many participants reported the need to be alert and continuously making such behavioral adjustments to some extent, minimize flares, some individuals expressed that continuous efforts to manage symptoms could disrupt their sense of normality and be a barrier to adjustment. To keep some sense of normality, some individuals (men and women) referred they made active efforts to *steer thoughts away from the illness* and its potential limitations. António (aged 41 years) referred that he only remembered having SLE when he took medications or had medical appointments *“I remember that I have lupus and that I am a sick person usually when I go to the hospital. (…) but my idea is always to live the day as if I were - in practice I am - a normal person.”*

However, when illness impairments prevent people from doing their most valued activities (e.g. going to the beach, doing sports), that is, when it *hampers the maintenance of normal life*, this can be very demotivating and scary, in the words of Emília (aged 38), “*I played sports often. The tiredness, the prostration, the failure to do things also caused me a lot of confusion. It affected me too..”* This is often associated with feelings of confusion, frustration, despair, anger or guilt.

In sum, the need to protect a sense of “having a normal life” was referred by most participants, regardless of their sex/gender, as being essential to a good adjustment to SLE, but that it was not always easy to achieve. Stress and sun exposure were seen as the major triggers of disease activity/flares, although only specific strategies to deal with the latter were mentioned. Being attuned to body changes and a balanced behavioral management of SLE signs and symptoms was seen as vital to keep a sense of normality and facilitate adjustment.

#### Theme 2. Others can be (un)helpful to SLE adjustment

Significant others (e.g. family, friends), other individuals with SLE, healthcare professionals or even strangers can have both a positive and negative impact on SLE adjustment. *Support from family and friends* was pointed out as important, especially emotional support (e.g. listening, understanding, being present) but also instrumental support (e.g. helping with domestic tasks such as cooking, washing clothes). Relationships with others can be helpful to maintain normality, manage work and vent, as expressed by Joana (aged 33 years), *“Because, yes, there are days when a person thinks, thinks a lot but, that*’ *s it, with the support of others, a person ends up forgetting, living normally.”* For one male participant, his children were seen as a source of motivation to keep going.

Conversely, some participants shared that relations with significant others can also originate stress and, hence, become a barrier to SLE adjustment, increasing its symptoms and burden. João (aged 55 years), for whom SLE causes severe disability, reported that some family members became more distant after the diagnosis and that this could also have impacted SLE activity, *“Family members distanced themselves from me, they didn*’ *t give me much support. These things also count a lot for a person’s illness.”* Lack of support or empathy and invalidation by significant others hampered illness adjustment.

Similarly, although relationships with healthcare professionals were highly valued when they were supportive, a few participants shared experiences of symptom invalidation or misunderstanding by their doctors. Healthcare professionals were also mentioned as the first and most valued source of health information, but only a few reported that their doctors were good information sources. Most individuals said that having detailed and reliable knowledge about the disease (e.g. symptoms, symptom triggers, comorbidities, medication, secondary effects) is paramount. However, most participants referred that healthcare professionals did not convey detailed information on SLE, or tended to use terms that were not understandable. As Marta (aged 34 years) illustrates:

*“(…) And [I ask doctors to] explain in detail how each medicine will influence the body. (…) I think these are always questions that doctors never want to answer, but I always ask this question, every time I have an appointment, but I never get an answer.”*

This leaves most individuals to a long and tentative self-learning process, which is often impaired by the lack of reliable and evidence-based information on the internet. The information they find is often difficult to apply to their specific case, hard to understand, or mainly negative, as shared by Raquel (aged 29 years): *“Because sometimes a person looks for information about the disease and only finds the bad things, the worst there is.”*

*Interactions with other individuals who lived the same experience* (e.g. same symptoms or a gestational loss because of SLE) were also mentioned. Some participants looked for these relationships online and felt that they were valuable resources for sharing information, experiences and ideas, comparing symptoms and finding “people like themselves.” Despite most participants mentioned they did not participate in support groups, they considered it to be a valuable resource, as Luísa (aged 24 years) referred *“I also think it would be very interesting to have this kind of support groups. (…) that*’ *s it, for a person to be able to talk to try to understand how the others are doing… ”*.

Furthermore, the *comparison with other cases of SLE* was potentially *motivational*, but it could also be *scary*, depending on the severity of the case. If the comparison target was living well with SLE, it increased participants’ optimism. Sometimes, doctors were the ones who told participants that most individuals lived well with SLE. In some instances, knowing about severe cases of SLE made participants feel grateful for not being in such bad shape. Knowing about other cases (especially if these were family members) could also be helpful by providing information about SLE and coping strategies, as stated by Alberto (aged 51 years):

*When I was diagnosed, I already had some of my father*’ *s experiences, although I didn*’ *t count on that. I didn*’ *t need a lot of information because I already had it (…).*

Conversely, if the comparison target was suffering a lot with SLE, it could be very scary and demotivating, especially if the participant saw it as one of her/his own possible futures. Emília (aged 36 years) referred that she had a family member that passed away because of SLE, so it was very scary when she received the diagnosis *“I just knew, and I was really scared because I had a picture of my cousin who had already passed away and I was really scared at the time”*. Participants mentioned that the same can happen in some SLE patient groups on social media, where the interaction can have a negative effect, not only by sharing unreliable information, but also by highlighting negative models and experiences.

*Illness public stigma* was also present on the narratives, mostly in terms of sarcastic, depreciative, or offensive comments about the visible SLE symptoms (e.g. rash, bruises) and body shame because of the weight gain. Invisible symptoms are often misunderstood, devalued or invalidated, as expressed by Manuela (aged 32 years), *“When we break an arm or a leg, people look at us and know, but this is something hidden, and people look and hardly believe it, and think that what we are saying is not quite like that….”* These experiences make individuals feel sad, demotivated and regretting having shared what they go through. Participants feel these comments also occur because SLE is not a well-known disease by the general population.

In sum, knowing and interacting with other individuals that live well with SLE and having supportive relationships with significant others and healthcare professionals are facilitators of SLE adjustment. However, if these other individuals are suffering a lot or are negative role models, if the relationships with others are stigmatizing or invalidating, these can be sources of stress hampering illness adjustment.

#### Theme 3 Overdoing and suffering the consequences versus making adaptations to maintain regular daily activity levels

Individuals with SLE expressed the need to deal with the ambivalence of having to do certain activities and recognizing that these entail an effort that is sometimes bigger than what they feel they should do. When they decide to engage in those activities there is a tendency to *overdo* them, especially in “good days” (i.e. when there is less pain or fatigue). Participants referred to that as “pushing it.” Consequently, they often experienced an increase in symptoms (e.g. fatigue, pain), leading to anger and frustration, which can be a barrier to adjustment, as described by António (aged 41 years) “*Therefore, when I overdo in terms of effort, it also has an influence on the lupus activity*.” However, deciding not to engage in those activities could lead to guilt. For Ana (aged 45 years) overdoing can emerge from a desire of seizing the day, *“On a day when we are well, we always go over our limits, because we want to enjoy it.”*

To *maintain a regular level of daily activity* most participants tried to define life priorities, regulate the time, speed and effort spent in the activity (e.g. negotiating deadlines), take breaks (i.e. resting or doing pauses), plan time for the unforeseen, regulate energy levels or take a nap. Most individuals stated that this was essential to keep being involved in activities despite fatigue, and it seemed to be associated with good adjustment, as shared by Sara (aged 49 years):

*I still do my job, without needing to ask for help. It*’ *s like I tell you - it*’ *s more the fatigue, but it*’ *s not so much that I don*’ *t do my work, I rest a little and do it again. Then I get home at the end of the day, I take a shower and that*’ *s it.*

Over engagement in activities can increase SLE symptoms but activity disengagement can also be a barrier to adjustment as it may lead to frustration. Strategies related to activity pacing can facilitate adjustment.

#### Theme 4. Self at odds versus becoming at peace with SLE

Receiving a diagnosis of a chronic condition such as SLE is threatening to one’s identity and difficult *to integrate in one’s self-concept.* For example, the need to take medication for life as a part of being “chronically ill” was recalled by most participants as being demotivating or sometimes revolting as this behavior permanently reminds them of “being chronically ill.” Being at odds with SLE is a barrier to SLE adjustment, as explained by Joana (aged 33 years) *“At first you get a bit angry, ‘Why did it happen to us?’.”*

*Negative thoughts* about the self but also the future were barriers to overcoming the challenges of SLE. Such pessimistic views often generated worry and anxiety about the future and the symptoms, hampering SLE adjustment. Some participants revealed that when they identified any SLE symptoms, they imagined the most pessimistic scenarios and ended up living in fear of getting sick again. As Raquel (aged 29 years) describes, these negative thoughts can make her panic over the smallest symptoms: “*Of course it*’ *s always that affliction, of getting sick again, of having something, any little thing I have, I panic, I*’*m already calling the doctor, that*’ *s it*.”

Conversely, with time, many individuals were able to *become at peace with SLE* and accept the illness as part of themselves. Being *optimistic* helped to envisage lupus as something that could be managed. Most participants stated that, with some adaptations, it could be possible to keep doing everything. The focus on solving problems, seizing the day and being resilient while maintaining a positive mindset were also helpful. The process of *integrating SLE in one’s self* and in one’s life was sometimes described as a process of “getting used to SLE,” and was marked by the acceptance of the disease and its impact on identity and daily life. This process seems to be associated with good illness adjustment.

In sum, *being at odds with SLE*—that is, the demotivation and anger caused by the perception of oneself as chronically ill, looking at the future with *pessimism*—is a barrier to SLE adjustment. However, with time, many individuals *became at peace with SLE*, which facilitated adjustment.

## Discussion

This study investigated the barriers and facilitators to SLE adjustment from the perspective of individuals with SLE. The results revealed four themes, two of them explored more in-depth by participants. The first theme stresses the desire and struggle to maintain a “normal life” through symptom management, whereas the second theme highlights how social relationships can either promote or hinder illness adjustment. The third theme stressed the importance of activity pacing and avoiding overdoing. The fourth theme highlighted the positive impact of being at peace with SLE and having an optimistic view about the future or, conversely, the negative impact of being at odds with SLE and being pessimistic. These themes depict factors that can work as either facilitators or barriers to SLE adjustment, and ranged from intra-individual characteristics, such as affective, cognitive and behavioral processes, to interpersonal factors, such as social support or stigma.

Stress, sun exposure and others’ invalidation, misunderstanding and stigma were the main perceived barriers to SLE adjustment. Stress was referred by participants as having potential to trigger a flare, which is supported by abundant evidence showing an association between distress and SLE symptoms (i.e. pain and fatigue; Azizoddin et al., 2019; Silva-Ribeiro et al., 2022; Sumner et al., 2020). Different strategies to avoid or reduce sun exposure were also mentioned by participants as a central self-management task to minimize SLE flares, and are frequent recommendations provided by healthcare professionals (Fanouriakis et al., 2019). Nonetheless, cross-sectional studies fail to find an association between sun exposure or photoprotection and disease activity (Abdul Kadir et al., 2018; Vilá et al., 1999).

Formal and informal negative relationships, characterized by invalidation and misunderstanding, can hamper adjustment (Hale et al., 2006; Sutanto et al., 2013). Formal relationships, such as doctor-patient relationships, can be supportive but also invalidating, as reported by SLE patients in previous studies (Petrocchi et al., 2022). The complexity and impact of this relationship is also referred by doctors, who considered doctor-patient communication critical to maintain trust and ensure appropriate care (Amsden et al., 2018). Healthcare professionals are also the first source of information, but when they are unavailable it is difficult to find reliable information, adapted to individual specificities (Neville et al., 2014). This unmet need of being informed by health care providers was also found in previous studies with SLE individuals (Hale et al., 2006).

Relationships with (significant) others can be stigmatizing. The stigma associated with visible SLE signs is in line with previous qualitative research, especially studies conducted with individuals with cutaneous lupus (Ogunsanya et al., 2018). Other qualitative studies also highlighted the relevance of invalidation and misunderstanding of invisible symptoms (Sloan et al., 2020). However, cross-sectional studies have scarcely addressed stigma in individuals with SLE, with a systematic review (Silva-Ribeiro et al., 2022) finding only one study showing an association with depression (Sehlo and Bahlas, 2013).

As for the factors associated with good SLE adjustment, participants stressed the importance of illness acceptance, managing symptoms to maintaining a sense of normality, maintaining positive interactions with others and regular daily activity levels. Individuals strive for a “normal life” through strategies that help them minimize SLE flares. Monitoring symptoms and adjusting behavior accordingly (e.g. changing medication, dieting or exercising) gives individuals a sense of control over SLE, allowing them to maintain their daily life and sense of normality (Oliveira et al., 2022; Petrocchi et al., 2022; Sutanto et al., 2013). However, over-attentiveness can disrupt the sense of normality and be identity threatening. Although the self-illness separation can sometimes impair self-management, it can also protect individual’s identity, reducing the fear of being consumed by the illness (Peters and Brown, 2022).

Significant others can support maintaining a normal life and activities. The positive influence familial relationships is frequently reported in other studies with SLE individuals (Petrocchi et al., 2022), being associated with lower depression (Narupan et al., 2022), anxiety (Zamora-Racaza et al., 2018) and fatigue (Burgos et al., 2009). Although the role of social factors on SLE adjustment has been less studied in quantitative research (Silva-Ribeiro et al., 2022), our participants strongly stressed their relevance. Sharing experiences and information with other individuals with SLE was valued by participants, as it can provide emotional support (Sloan et al., 2020). However, social comparisons lead to both positive and negative effects, depending on whether these were downward or upward (Brennan and Creaven, 2016).

Like in previous studies (Petrocchi et al., 2022; Sutanto et al., 2013), the maintenance of regular activity levels was seen as key to maintaining a normal life, which required diverse adaptations (e.g. define priorities, take breaks, negotiate deadlines) and avoiding overdoing. This stresses the relevance of engaging in adaptive activity patterns for SLE adjustment (i.e. regular and consistent behavioral patterns that occur in daily activities or occupations; Bendixen et al., 2006), a topic that has been totally neglected in the SLE literature in the past years (Silva-Ribeiro et al., 2022). Nevertheless, the role of activity patterns has been amply studied with other chronic pain conditions, suggesting that patterns of avoidance or excessive activity are often associated with poorer physical and psychological function (Andrews et al., 2012), which is consistent with our results.

As in the context of other chronic conditions, SLE adjustment is improved with acceptance (Peters and Brown, 2022), optimism, resilience and focus on problem-solving, with illness representations often changing over time (Goodman et al., 2005). This is consistent with previous qualitative (Sutanto et al., 2013) and cross-sectional studies (Silva-Ribeiro et al., 2022) showing that resilience related factors (e.g. optimism, hope) and adaptive illness- and treatment-related beliefs (e.g. positive illness perceptions) have a strong association with better psychological health and quality of life. These results highlight the relevance of individuals’ beliefs about SLE on illness adjustment (Goodman et al., 2005; Leventhal et al., 1992, 2003).

### Limitations and future directions

This study has several limitations. First, it was not possible to explore sex/gender differences because most of participants were women. This can be relevant to study in future research to understand how facilitators and barriers of adjustment can differ by sex/gender. Second, only white, low middle class SLE Portuguese individuals participated in the study. As social and cultural background or environmental factors (e.g. socioeconomic status, availability of health care; Moss-Morris, 2013) can impact illness adjustment, future studies are needed with other more diverse populations. Third, some individuals had other chronic diseases, so some of the processes shared by participants may not be exclusive of SLE adjustment, as it was difficult for some of those participants to distinguish the impact of SLE from other chronic illnesses. Moreover, seven participants have self-reported their diagnosis, so we could not be entirely sure they met lupus diagnosis criteria (Aringer et al., 2019). Finally, despite individual interviews being a good method to unveil personal perspectives, results can be influence by social desirability and interviewer bias. Despite these limitations, participants presented a big range of ages and disease durations, allowing to tap into perspectives of individuals in different phases of the life and illness cycle. Our findings have important implications, namely the identification of factors that can be targeted by future interventions, such as stress, social support, stigma, access to information, illness representations and activity patterns. Stress reduction interventions, integrating mindfulness or cognitive-behavioral strategies, have shown promising results with individuals with SLE (Navarrete-Navarrete et al., 2010), so there is a potential for further intervention development in this area. Social factors should be considered on future intervention development. For example, a psychoeducational intervention to promote social support with SLE individuals showed positive results (Karlson et al., 2004). Furthermore, the positive and negative impact of comparison with other patients can inform group intervention development (van Dam et al., 2005). It can also give a new perspective to the development of personas for digital interventions, contributing to increase identification and motivation, but also providing awareness of negative comparisons’ potential (Bartels et al., 2023). As SLE stigma is common, psychoeducational interventions promoting self-advocacy and family and public education are needed (Ogunsanya et al., 2018; Sutanto et al., 2013). The difficulty to access reliable information also stresses the need to develop educational resources with information validated by healthcare professionals, which can be made widely and quickly available (Neville et al., 2014). To improve illness representations, future interventions should encourage exploring personal perspectives about the illness. For example, cognitive-behavioral therapy strategies have shown to be effective with other chronic conditions (Shan et al., 2022), and Acceptance and Commitment Therapy allows an exploration of meaningful life directions despite illness (Prevedini et al., 2011). Activity pacing, including some of the strategies shared by the participants (e.g. defining priorities, taking breaks, negotiating deadlines; Esteve et al., 2017; McCracken and Samuel, 2007), has shown promising results as an intervention strategy to regulate activity patterns, and decrease the fluctuation between peaks of overactivity and sedentary behavior (Guy et al., 2019). Considering the centrality of having a “normal life,” it is important that healthcare professionals undertake regular assessment of illness interference, helping patients to find treatment options and psychosocial interventions that can maintain or restore their sense of normality. This idea is supported by recent clinical recommendations to improve the quality of life of individuals with SLE (Schlencker et al., 2022).

Future research should focus on some of the facilitators/barriers to SLE adjustment highlighted in this study in more detail. For instance, considering the strong impact of stress on SLE activity and adjustment (Azizoddin et al., 2019; Sumner et al., 2020) it will be important to further investigate strategies that SLE individuals use to manage stress and what needs and preferences they have on this matter. Although researchers have not yet directly investigated the role of activity patterns in SLE adjustment, our findings suggest this may also be an important avenue for further research, with prospective designs. Social factors should also be investigated with more detail in the future, including different aspects such as preferences for interaction with others, satisfaction and perceived availability of support (Bernardes et al., 2017) regarding different types of relationships (e.g. significant others, healthcare professionals, or unknown).

In conclusion, this study highlighted that SLE adjustment can be promoted by maintaining a sense of “normal life” and optimism, for which the ability to maintain the engagement in meaningful activities, having reliable illness-related information and supportive relationships are key. Conversely, trying to overdo things, having a pessimistic outlook, feeling unsupported or stigmatized by others, and lacking reliable information are risk factors to SLE adjustment to be addressed in future interventions to promote individuals’ quality of life.

## Supplemental Material

sj-docx-1-hpq-10.1177_13591053241296190 – Supplemental material for Maintaining a sense of normality with the help of others: Lived experiences of facilitators and barriers to Lupus adjustment Supplemental material, sj-docx-1-hpq-10.1177_13591053241296190 for Maintaining a sense of normality with the help of others: Lived experiences of facilitators and barriers to Lupus adjustment by Sofia Silva-Ribeiro, Sónia F Bernardes, Marta M Marques and Cristina A Godinho in Journal of Health Psychology
