# Supplementary material for: Maintaining a sense of normality with the help of others: Lived experiences of facilitators and barriers to Lupus adjustment
Source: J Health Psychol. 2024 Nov 22;30(10):2767–80. doi: 10.1177/13591053241296190 (PMC12381384; doi:10.1177/13591053241296190)
Supplement: sj-docx-1-hpq-10.1177_13591053241296190 – Supplemental material for Maintaining a sense of normality with the help of others: Lived experiences of facilitators and barriers to Lupus adjustment [file sj-docx-1-hpq-10.1177_13591053241296190.docx]

**Supplementary File 1. Interview Scrip**

**Table 1.** Interview schedule structure, topics and questions

| **Main topics** | **Questions** | **Example of exploratory questions** |
| --- | --- | --- |
| Section 1- Ice-breaking questions to explore:   - the experience of receiving a SLE diagnosis; - illness progress since diagnosis; - treatments followed since diagnosis. | - *Could you introduce yourself and tell me about the time you got your SLE diagnosis?* - *How has your illness evolved since then?* - *What treatments do you currently follow?* | - *Do you still have those symptoms that you had at diagnosis?* - *What symptoms do you have nowadays?* |
| Section 2 - The aim of this section was to explore:   - the impact of SLE in different life domains; - facilitators and barriers that individuals considered that could be associated with better or poorer adjustment. | - *What impact do you feel that Lupus has on your life? And in what areas?* - *What factors/ situations/ conditions do you consider that, despite the illness, have helped (or made it difficult for) you to have a better quality of life?* | - *And what about other factors?* - *And what about stress, for example?* |
| Section 3 – Included questions about the needs and preferences regarding a digital intervention to improve SLE adjustment. | - *Would you be willing to use a digital intervention to help you make some changes that could improve your quality of life?* - *How do you imagine using this intervention?* | - *For example, would you like to have reminders for your appointments?* - *Would you prefer to use this intervention on your mobile or computer?* |

**Supplementary File 2. Participants characterization**

**Table 2.** Participants’ sociodemographic and clinical characteristics

| Participant Alias | Self-identified gender | Age (years) | Disease duration | Other illness | Employment status^1^ |
| --- | --- | --- | --- | --- | --- |
| Maria | Women | 44 | 3 | Antiphospholipid Antibody Syndrome | E |
| Joana | Women | 33 | 13 | - | E |
| João | Men | 55 | 8 | - | UW |
| Ana | Women | 45 | 12 | - | E |
| Sara | Women | 49 | 5 | Hypertension | H |
| Marta | Women | 34 | 20 | Hypothyroidism | S |
| Luísa | Women | 24 | 5 | Psoriatic Arthritis, lupus panniculitis | S |
| Rita | Women | 40 | 18 | - | E |
| Manuela | Women | 32 | 17 | Rheumatoid arthritis | E |
| Rosa | Women | 36 | 17 | - | E |
| Emilia | Women | 38 | 12 | - | U |
| António | Men | 41 | 18 | - | E |
| Paula | Women | 45 | 18 | - | E |
| Alberto | Men | 51 | 21 | Renal insufficiency | E |
| Raquel | Women | 29 | 7 | - | E |
| Ema | Women | 38 | 8 | Hypothyroidism | H |

1. Employment status: S = student; E = employed; U = unemployed; UW = unable to work; H = Housekeeper
